# A method to extract slip system dependent information for crystal plasticity models

**DOI:** 10.1016/j.mex.2022.101763

**Published:** 2022-06-20

**Authors:** Dylan Agius, Abdullah Al Mamun, Christopher Truman, Mahmoud Mostafavi, David Knowles

**Affiliations:** a Solid Mechanics Research Group, Department of Mechanical Engineering, University of Bristol, United Kingdom; b Nuclear Futures Institute, Bangor University, Gwynedd LL57 2DG, United Kingdom

**Keywords:** Crystal plasticity, Grain size effect, Slip distance, Grain boundary, Misorientation, Slip system interaction

## Abstract

A tool to implement a length scale dependency to classical crystal plasticity simulations is presented. Classical crystal plasticity models do not include a size effect; therefore, the size of the grain does not influence the simulated deformation. Classical crystal plasticity advancements have been through the inclusion of stress or strain gradient based constitutive models to improve the simulation of length scale dependent deformation. However, this tool presents an alternative to implementing a length scale, where the influence of slip pile-up in the form of dislocations at grain boundaries as a potential to explaining the Hall-Petch effect in materials. This is achieved by calculating the slip distance in adjacent grains for each slip system, by assuming the total slip length spans the grain in the slip direction. These calculations can occur in two ways. The first is the analysis occurs at the start of the simulation, therefore, only occurs once. If this approach is used, the computational cost of this tool is minute. However, if the simulations consider large deformations, during which it is expected that the grains are going to undergo large rotations, then it would be advantageous to the have the tool recalculate the information during the analysis. Consequently, the computational cost would depend on the resolution of the modelled geometry, the number of grains, and the number of slip systems. The tool also provides a capability to develop constitutive models based on complex grain boundary features which can be implemented in classical crystal plasticity models and gradient based crystal plasticity models. The described calculation process is implemented through a Fortran subroutine, which has been designed to be easily used in crystal plasticity simulations. The presented tool also includes Python code designed to link with microstructures built using DREAM.3D to extract the required input data to the Fortran subroutine.

The proposed tool is not limited to classical crystal plasticity formulations, instead the data extracted and outputted from the Fortran subroutine can be used to serve alternative purposes in both stress and strain gradient crystal plasticity models.

The proposed tool can be modified to extract additional data to that presented.

The slip distance in the adjacent grain, the distance from the grain boundary of the current calculation point, and the interaction between slip systems between grains can be used in any crystal plasticity constitutive models.

## Graphical abstract

**Figure:**
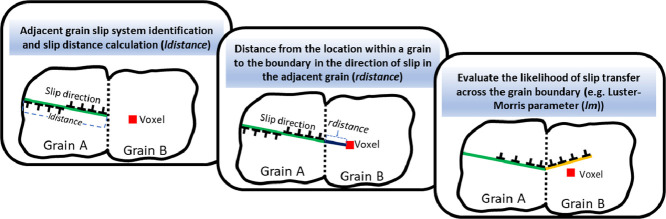

**Specifications table**

**Table:** 

Subject Area:	Materials Science
More specific subject area:	*A tool to implement a length scale dependency to crystal plasticity simulations*
Method name:	*LengMorph: a tool to add a length scale dependence to crystal plasticity simulations.*
Name and reference of original method:	*N.A.*
Resource availability:	*The Fortran subroutine and Fortran example program files will be made available in the supplementary documentations. All other materials can be found from the following GitHub repository:*https://github.com/DylanAgius/LengMorph.git*.*

## Method details

In the following sections, details of an algorithm to implement a length scale dependency to classical crystal plasticity models is outlined in detail. The approach is based on calculating slip distances for each slip system at a voxel/element using the geometry and orientation of the closest adjacent grain. The proposed approach is based on the theory of the underlying mechanisms of the Hall-Petch effect being associated with dislocation pile-ups at grain boundaries [2], [3], [4], [5], [6], [7], [8], [9]. The simulated incompatibility between grains is modified through the critical resolved shear stress (CRSS) to reflect the influence of pile-ups in adjacent grains. It must be noted that the current tool has been generalised to voxel-based representative volume elements (RVE); therefore, it cannot currently be used with tetrahedral discretised RVEs.

**Fig. 1:**
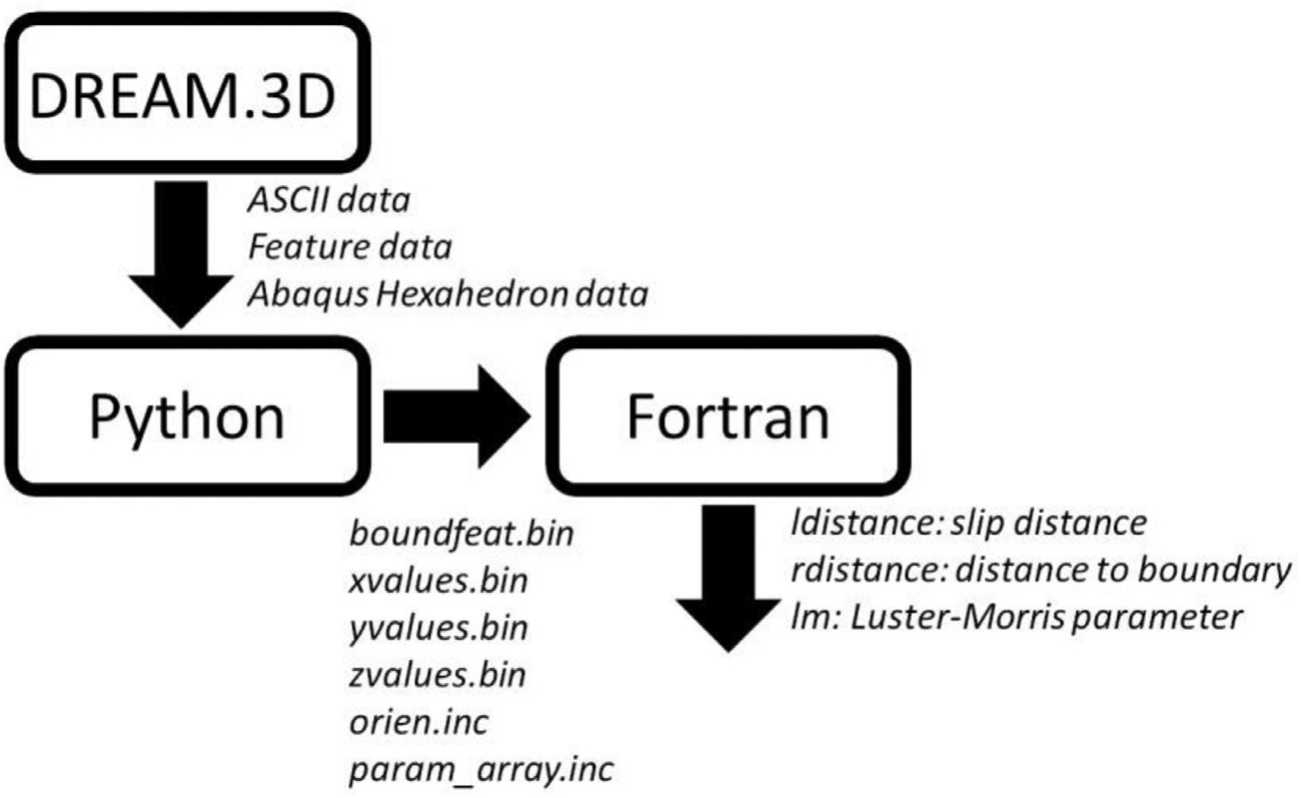
Integration of software to construct the input data required for the Fortran length scale code which outputs information (ldistance, rdistance, lm) to be applied in crystal plasticity constitutive models found in [1].

In the present work the code used to construct the necessary input data (in Python [17]) with the code used to calculate the slip distance (in Fortran) are outlined. An additional output of the Fortran code is the Luster-Morris parameter [11] which can also be used in crystal plasticity constitutive models as demonstrated in [1]. RVEs employing the length scale size dependence can be synthetically built using DREAM.3D [10], with details on the DREAM.3D constructed grains fed into the Python code. The integration of these codes is schematically shown in Fig. 1.

The specifics of how the length scale dependent subroutine presented here integrates with classical crystal plasticity theory can be found in [1]. The tools presented facilitates implementation in both finite element methods and spectra methods based on Fast Fourier Transforms. All materials needed to implement the proposed approach can be found at [12], which includes a DREAM.3D pipeline to construct the required information to be fed into the Python program used to create the input data and a standalone Fortran program which demonstrates how the Fortran code functions.

### Extracting and processing grain data (DREAM.3D and python)

**Fig. 2:**
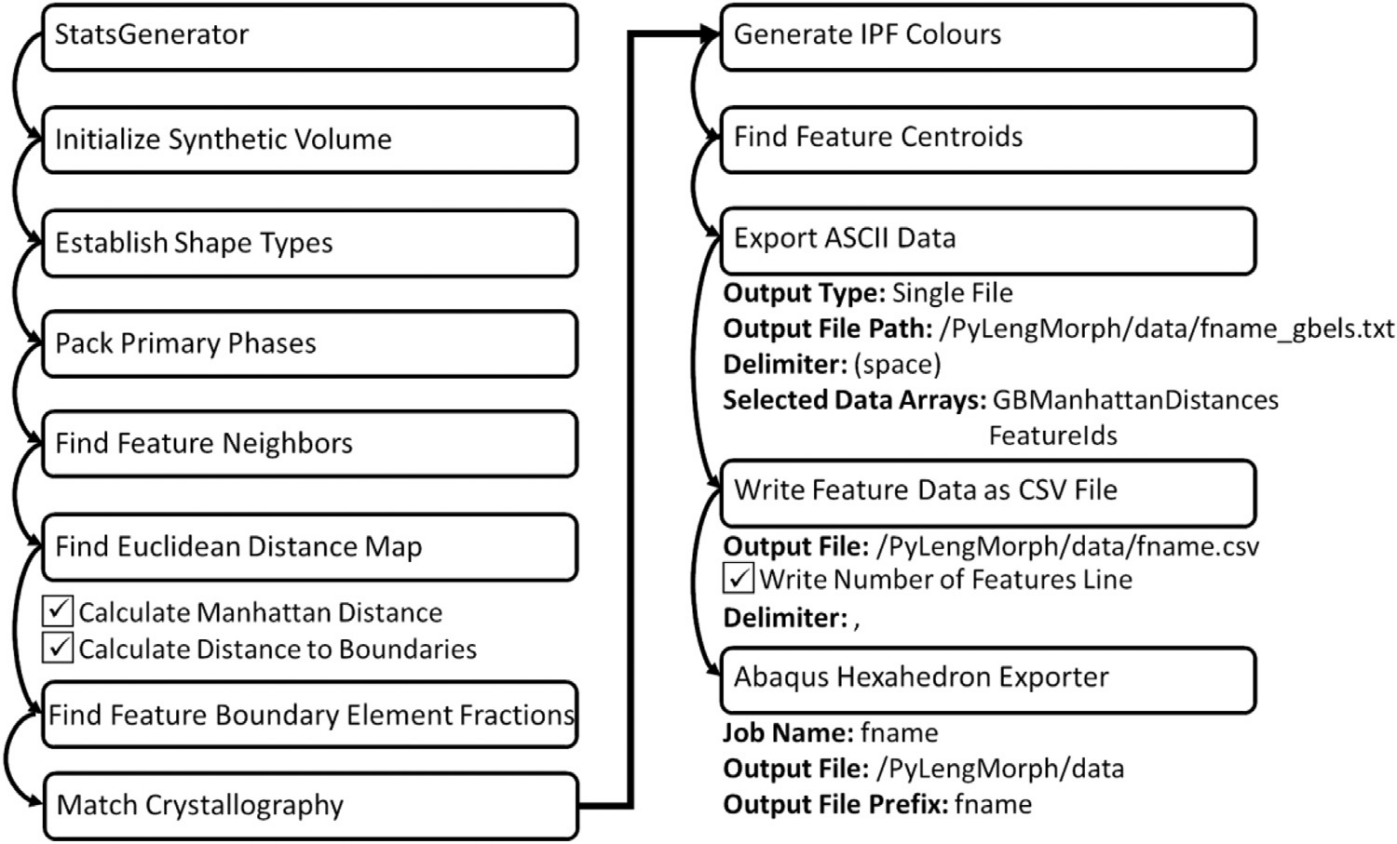
Example DREAM.3D pipeline included the additional filters required to extract information for the Python code.

One effective tool to create RVEs for different microstructures is DREAM.3D. Using available filters in the DREAM.3D pipeline, information on the features of the generated grains can be extracted. This includes the distance of each voxel to the grain boundary, the centroid, and orientation of each grain. An example minimum filter pipeline to create an RVE is provided in Fig. 2, which includes additional filters used to extract information needed as inputs to the Python code in the present work. This example DREAM.3D pipeline can be found at [12].

**Fig. 3:**
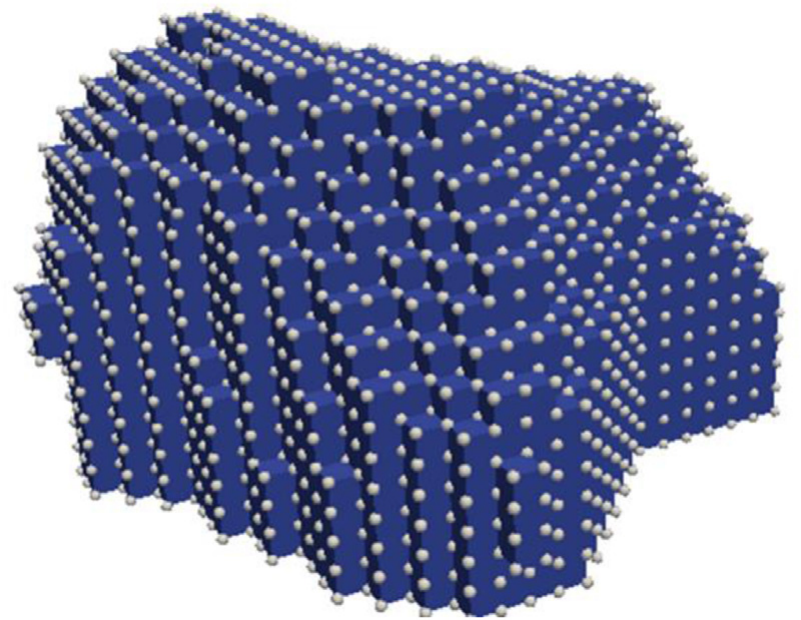
Nodes on the boundary of a grain. The location in the global coordinate system and the grain IDs of each grain which shares each node used as input data to the Fortran code.

The DREAM.3D extracted data is used in the Python code to construct matrices containing information of nodes on the boundary of every grain in the RVE as visualised in Fig. 3. This information includes the location in x,y, and z coordinates (in the global coordinate system), and the grain IDs of all grains which share these boundary nodes.

**Fig. 4:**
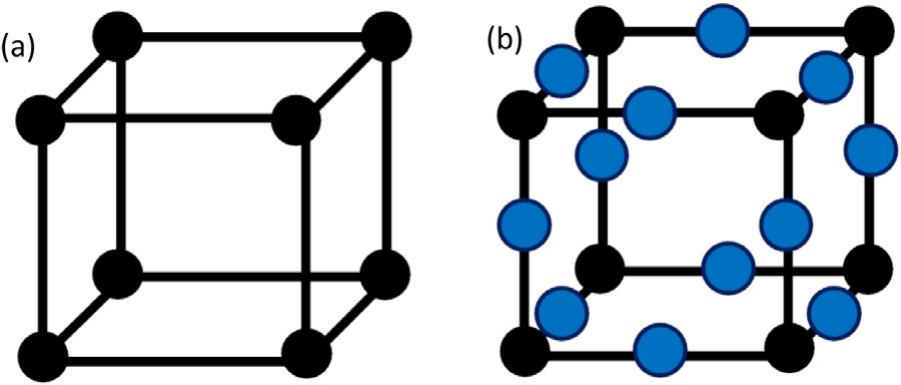
Difference in the number of nodes defining a voxel/element where (a) is the minimum number of nodes, and (b) is the maximum number of nodes, where the additional nodes are given in shaded blue.

The Python code makes use of the NumPy [2] and pandas data analysis [3] libraries. An option is provided to the user to select two possibilities for the density of nodes on the boundary of the grain. A minimum number of nodes defining the grain boundary can be used which refers to the node locations provided in Fig. 4(a) for an example voxel/element. Alternatively, there is the possibility to increase the number of nodes to what can be observed in Fig. 4(b). The advantage of increasing the number of nodes on the grain boundary is it ensures a more accurate vector selection from which the slip distance can be calculated. This is because there is a greater number of possible vectors which can be created between the current location to the boundary, increasing the probability of selecting a vector in the closest possible orientation to the slip direction. However, doing so will increase the computational time of the analysis since there are a greater number of nodes to cycle through. Therefore, it is up to the user to choose the preferred option.

The code used to construct the required input matrices can be downloaded from [12] and installed by navigating to the folder *PyLengMorph* before using the install pip command. Once installed, the function *grainboundary* can be used by applying the following convention, (import PyLenghMorph.grainboundary(loc=‘/path/to/folder’, file=‘fname’, nodeinc=True,abq=True)

Four inputs are required to use this function:

1 loc – path to the *data* folder which contains all the data generated by DREAM.3D (as described in Fig. 2).
2 Fname - name of the files generated by DREAM.3D as indicated in Fig. 2 (filters: *Export ASCII, Export Feature Data CSV File, Abaqus Hexahedron Exporter*). These files should be located in */data.*
3 Nodeinc - either *True* or *False* can be used, where *False* results in each boundary voxel defined by the minimum number of nodes (as demonstrated in Fig. 4(a)), and *True* the maximum number of nodes (as demonstrated in Fig. 4(b)).
4 abq – either *True* or *False* can be used, where *True* results in the creation of Fortran INCLUDE files which can be used with a Fortran fixed-form source, while *False* results in an INCLUDE file to be used with a Fortran free-form source.

**Fig. 5:**
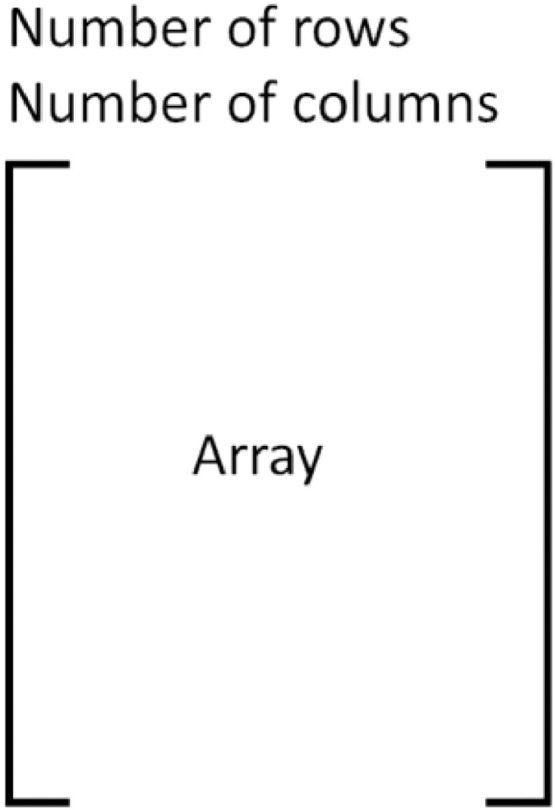
Structure of the xvalues.bin, yvalues.bin, zvalues.bin, boundfeat.bin, and el_centroid.bin files, where ‘Number of rows’ is the integer of the rows of the extracted matrix, and ‘Number of columns’ is the integer of the columns of the extracted matrix.

After calling *grainboundary*, the input matrices are written to five different binary files with the structure of the file provided in Fig. 5, where *Number of rows/columns* refers to the dimensions of the matrix which are located on the first and second line of the file, respectively. The matrix can be read at the very start of a crystal plasticity analysis.

The five binary files created by *grainboundary* are as follows*:*

• Xvalues.bin, yvalues.bin, zvalues.bin – each file contains the x, y, and z coordinates of each boundary node in the global coordinate system.
• Boundfeat.bin – the grain ID of the grain which each shares the boundary node.
• El_centroid.bin – the centroid coordinates of all voxels/elements within the RVE.

**Fig. 6:**
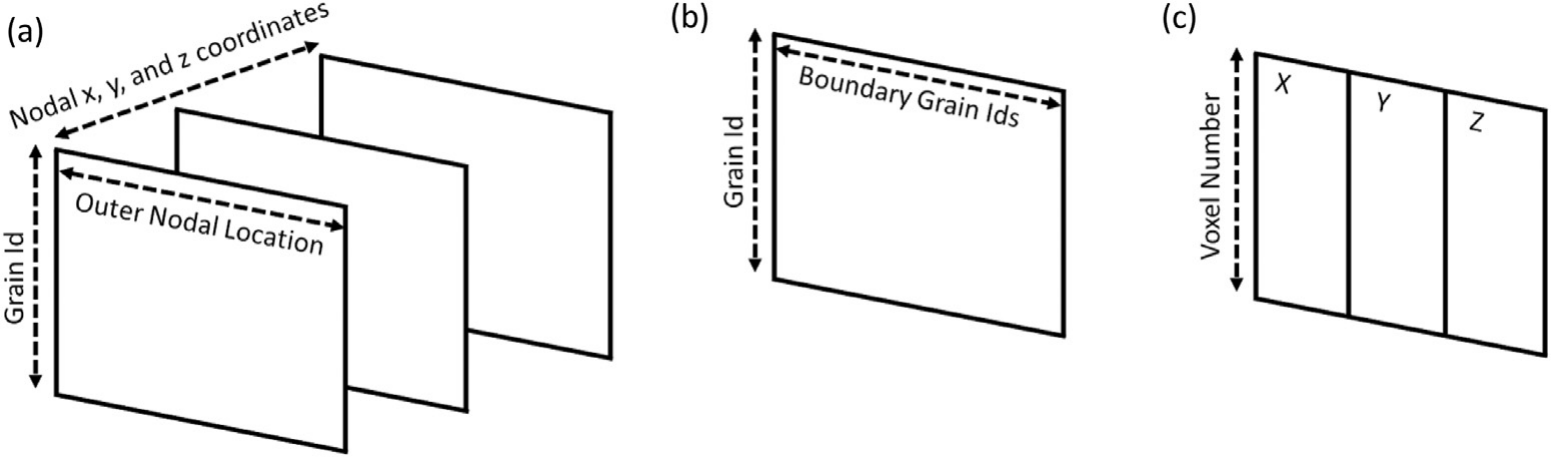
Schematic of the matrices used in the analysis to implement in the grain size-morphology modification. (a) The row index represents the grain ID of interest, while the columns contain the coordinates of the surface nodes belonging to this grain. (b) A matrix where the row index represents the grain of interest, and the columns corresponding to grain IDs of the grains on the surface of the grain of interest. (c) A matrix of voxel/element centroids where the row index corresponding to the voxel/element number while the columns are the x,y,z coordinates.

The structure of these matrices is provided in Fig. 6. Fig. 6(a) corresponds to the matrices contained in xvalues.bin, yvalues.bin, zvalues.bin, where the row index corresponds to the grain ID of the grain the surface nodes belonged to. Fig. 6(b) corresponds to the matrix contained in boundfeat.bin. Each row corresponds to the grain ID sharing each boundary node. The row index once again refers to the grain ID which contains these boundary nodes. Fig. 6(c) corresponds to the matrix contained in el_centroid.bin where each row is the voxel/element centroid coordinates.

A Fortran INCLUDE file (orien.inc) is also created, which contains an array of all Euler angles for each grain. This information is used to create the array *oriensh* which is an input to the Fortran subroutine described in the following sections. The crystal plasticity code can be directed to this file using the convention,

INCLUDE ‘orien.inc’

Finally, if *abq=True*, an additional INCLUDE file is created (*param_array.inc*). This file contains the sizes of the matrices in the binary files. This is an important INCLUDE file if Abaqus is to be used. The binary files can be read in at the very start of the analysis using the subroutine *UEXTERNALDB,* with the dimensions of these matrices provided in *param_array.inc.* This ensures the matrices can be shared between subroutines using a *COMMON BLOCK*. An example of how this is implemented is provided in the example subroutine *Example_UEXTERNALDB.for* which can be found at [12].

### Slip distance and misorientation calculation (Fortran)

In this section, the calculation approach is detailed along with the corresponding Fortran code (the nomenclature of which is listed in Table 1) which implements the presented approach. Outlined in this section are the details contained in the Fortran subroutine which is used to calculate the slip distance and Luster-Morris parameter. In the following mathematical explanation, α is used to represent a slip system since the calculation approach occurs for each slip system.

**Table 1:** Nomenclature (listed in order of appearance) used in the Fortran code used to demonstrate how the presented mathematical formulations used in the calculation process are implemented.

Input/output names	Description
feature	The grain ID which contains the current voxel/element.
arraysize	Maximum number of nodes on the surface for each grain.
nodex(:,arraysize), nodey(:,arraysize), nodez(:,arraysize)	x, y, and z-coordinates of all nodes on the surface of the current grain.
coords(3)	Coordinates of the centroid of the current voxel (FFT software) / coordinates of the node of the current element (FE software)
vect(:,:)	x, y, and z components of all vectors drawn from the voxel/element to the boundary nodes.
minindexing	Index of the location of the minimum Euclidean distance.
grainb	Grain ID of the closest adjacent grain.
slpdir(:,:), slpplane(:,:)	Slip directions and slip planes in the local (crystal) coordinate system.
oreinsh(:,:)	Array of Euler angles for each grain.
totalrot(3,3)	Rotation matrix to rotate the local slip closest grain to the global coordinate system.
slpdirrotate(:,:),slpplanrotate(:,:)	Slip direction and slip planes in the global coordinate system.
featureboundsnodes(:,:)	x, y, and z coordinates of all shared boundary nodes.
btotal(:,:)	Vector from the voxel/element to the shared boundary.
normarray(:)	Magnitudes of all vectors (btotal).
normslp(:)	Magnitudes of slip direction (slpdir).
minang0(:,:), minang180(:,:)	Minimum angles.
minangleval(:), minangleval180(:)	Index of the minimum angles.
minangleactual(:), minangleactloc(:)	Overall minimum angle and the corresponding index.
boundnodegrainb(:)	The index of the node on the shared boundary.
rnodes(:,3)	Coordinates of the node on the shared boundary.
nodesnotindex(:)	Index of nodes in grain B not at the interface of grain A and grain B.
vectr(:,:)	Vectors form the current voxel/node to the grain boundary nodes in grain B (adjacent grain).
vectrnorm(:)	Magnitude of vectr(:,:) for each slip system.
lxtotal(:,:,:)	Vectors from the grain boundary node to the grain boundary nodes in grain B (adjacent grain) for each slip system.
lxnorm(:)	Magnitude of vectors lxtotal for each slip system.
btotal(:,:)	Vector from voxel/element to the shared boundary.
normarry(:)	Magnitudes of all vectors (btotal).
normslp(:)	Magnitudes of slip direction (slpdir).
minxval0(:,:), minxval180(:,:)	Index of the minimum angles of each array.
minxval0act(:,:), minxval180act(:,:)	Minimum angles of arrays.
minxangle(:)	Overall minimum angle for each slip system.
minxangleact(:)	Overall minimum angle index for each slip system.
lxnodesindex	Index of the node at the boundary of grain B.
lxnodes(:)	Coordinates of the identified node on the boundary away from the grain A and B interface.
**Subroutines**	**Description**
eulercosmatrix	Constructs the rotation matrix base on the Euler angles.
enorm	Calculates the magnitude of vectors.

### Calculation approach

Starting from the current voxel/element at which the calculation is being conducted, the grain ID that the voxel/element belongs is determined. This information can be fed in as a property definition of the grain. Once this is known, the matrices containing the grain IDs and nodal coordinates are used. For the current voxel (or integration point if finite elements are being used) ($P_{vox/el}$), the Euclidean distance (d) for each node ($Q_{node}$) on the boundary of the grain containing the current voxel/element is calculated. The adjacent grain is determined from the index ($d_{index}$) of the minimum Euclidean distance,

(1) $$d^{\alpha }{\left(P_{vox/el},Q_{node}\right)}_{i}=\sqrt{\sum_{k=1}^{n}{\left(Q_{node/k}-P_{vox/elk}\right)}^{2}},where\;D^{\alpha }\left(P_{vox/el},Q_{node}\right)=\left\{d^{\alpha }{\left(P_{vox/el},Q_{node}\right)}_{i}\;i\in Z^{+}\right\}$$

(2) $$d_{index}^{\alpha }=\text{argmin}\left(D^{\alpha }\left(P_{vox/el},Q_{node}\right)\right)$$

Once the minimum Euclidean distance is determined, the nearest grain can be determined from the inputted matrices. Using the column index of the node determined to be the shortest distance, the second matrix Fig. 6(b) is used to determine the grain ID that this boundary is associated with.

Once this boundary grain is known, the Euler angles ($\varphi_{1}$, Φ, $\varphi_{2}$) defining its orientation within the global coordinate system can be extracted. This information can be extracted from the matrix defined in *Orien.inc*. Once the Euler angles are determined they are used to form a rotation matrix (R),

(3) $$R=\left[\begin{array}{ccc} \cos\varphi_{1}\cos\varphi_{2}-\cos\Phi \sin\varphi_{1}\sin\varphi_{2} & \sin\varphi_{1}\cos\varphi_{2}+\cos\Phi \cos\varphi_{1}\sin\varphi_{2} & \sin\Phi \sin\varphi_{2}\\ -\cos\varphi_{1}\sin\varphi_{2}-\cos\Phi \sin\varphi_{1}\cos\varphi_{2} & \cos\Phi \cos\varphi_{1}\cos\varphi_{2}-\sin\varphi_{1}\sin\varphi_{2} & \sin\Phi \sin\varphi_{2}\\ \sin\Phi \sin\varphi_{1} & -\sin\Phi \cos\varphi_{1} & \cos\Phi \end{array}\right]$$

Using the rotation matrix, the slip direction ($S^{\alpha }$) and slip normal ($N^{\alpha }$) in the local coordinate system can be rotated to the global coordinate system ($s^{\alpha }$, $n^{\alpha }$),

(4) $$s^{\alpha }=R^{T}S^{\;\alpha };n^{\alpha }=R^{T}N^{\alpha }$$

This calculation occurs for both grain A ($s_{A}^{\alpha }$ and $n_{A}^{\alpha }$) and B ($s_{B}^{\alpha }$ and $n_{B}^{\alpha }$).

**Fig. 7:**
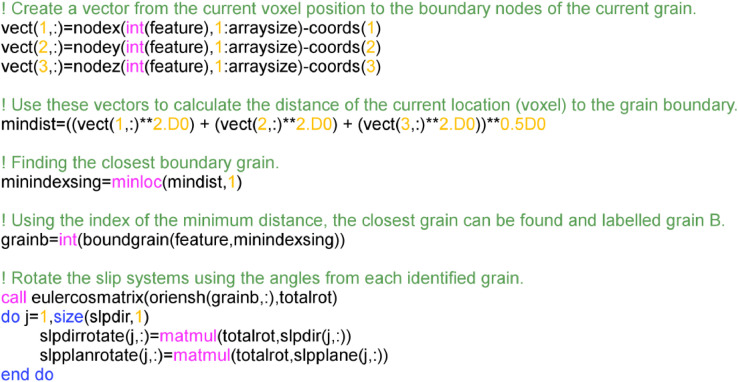
Fortran code used to calculate the closest grain to the current voxel/element based on the calculated Euclidean distance.

The source code for how this implemented for grain B is provide in Fig. 7 implemented in Fortran language.

**Fig. 8:**
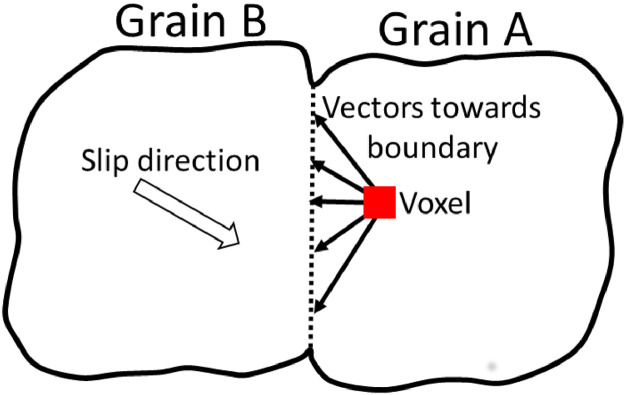
A schematic demonstrating the approach to find the most suitable nodes on the shared boundary which results in the same orientation as the slip direction in the adjacent grain (grain B).

Once the closest grain boundary is determined, the vectors from the current voxel/element can be formed towards each of the nodes on at the shared boundary. This is done to determine which node at the shared boundary ensures the formation of a vector which is deemed to be in the same orientation as the slip directions in the adjacent grain. This is shown schematically in Fig. 8.

To achieve this, the coordinates of the feature boundary nodes are determined, which are then used with the voxel/element coordinates to find the corresponding vector ($a{{}_{bound}^{\alpha }}_{i},\;\text{where}\;A_{bound}^{\alpha }=\left\{a{{}_{bound}^{\alpha }}_{i}|i\in Z^{+}\right\}$). These vectors are compared to the slip directions (for each slip system α) in the adjacent grain ($s_{B}^{\alpha }$) by calculating the angle between them ($\theta {{}_{diff}^{\alpha }}_{i}$),

(5) $$\theta_{diff\;i}^{\alpha }=\cos^{-1}\frac{a{{}_{bound}^{\alpha }}_{i}\cdot s_{B}^{\alpha }}{\left|a{{}_{bound}^{\alpha }}_{i}\right|\left|s_{B}^{\alpha }\right|}$$

The angle is calculated for all vectors resulting a set of values. The boundary vector and slip vector are parallel if the angle between them is 0 and 180°, therefore, the minimum for these two cases are extracted,

(6) $$\theta_{0}^{\alpha }=\left(\Theta_{diff}^{\alpha }\right);\;\theta_{180}^{\alpha }=\left(\left|\Theta_{diff}^{\alpha }-180\right|\right),\;\text{where}\;\Theta_{diff}^{\alpha }=\left\{\theta_{diff_{i}}^{\alpha }\;i\in Z^{+}\right\}$$

The index of the location of the minimum angles are also stored for later use,

(7) $$\theta_{0,index}^{\alpha }=\text{argmin}\left(\Theta_{diff}^{\alpha }\right);\;\theta_{180,index}^{\alpha }=\text{argmin}\left(\left|\Theta_{diff}^{\alpha }-180\right|\right)$$

The final minimum angle for each slip system ($\theta_{min}^{\alpha }$), and therefore the corresponding boundary node can be determined by comparing the two minimums to find the total minimum,

(8) $$\theta_{min}^{\alpha }=\left(\theta_{0},\theta_{180}\right)$$

Using the index of the location within the set at which the minimum is located, the corresponding vector can be extracted,

(9) $$a_{bound}^{\alpha }=a{{}_{bound}^{\alpha }}_{k},\;\text{where}\;k=\theta_{0,index}^{\alpha }\vee \theta_{180,index}^{\alpha }$$

The magnitude of this vector ($a_{bound}^{\alpha }$) is also calculated to extract the distance from the current voxel to the boundary. This is an additional output not utilised in the crystal plasticity constitutive models in [1] but can be used is in alternate crystal plasticity constitutive models.

**Fig. 9:**
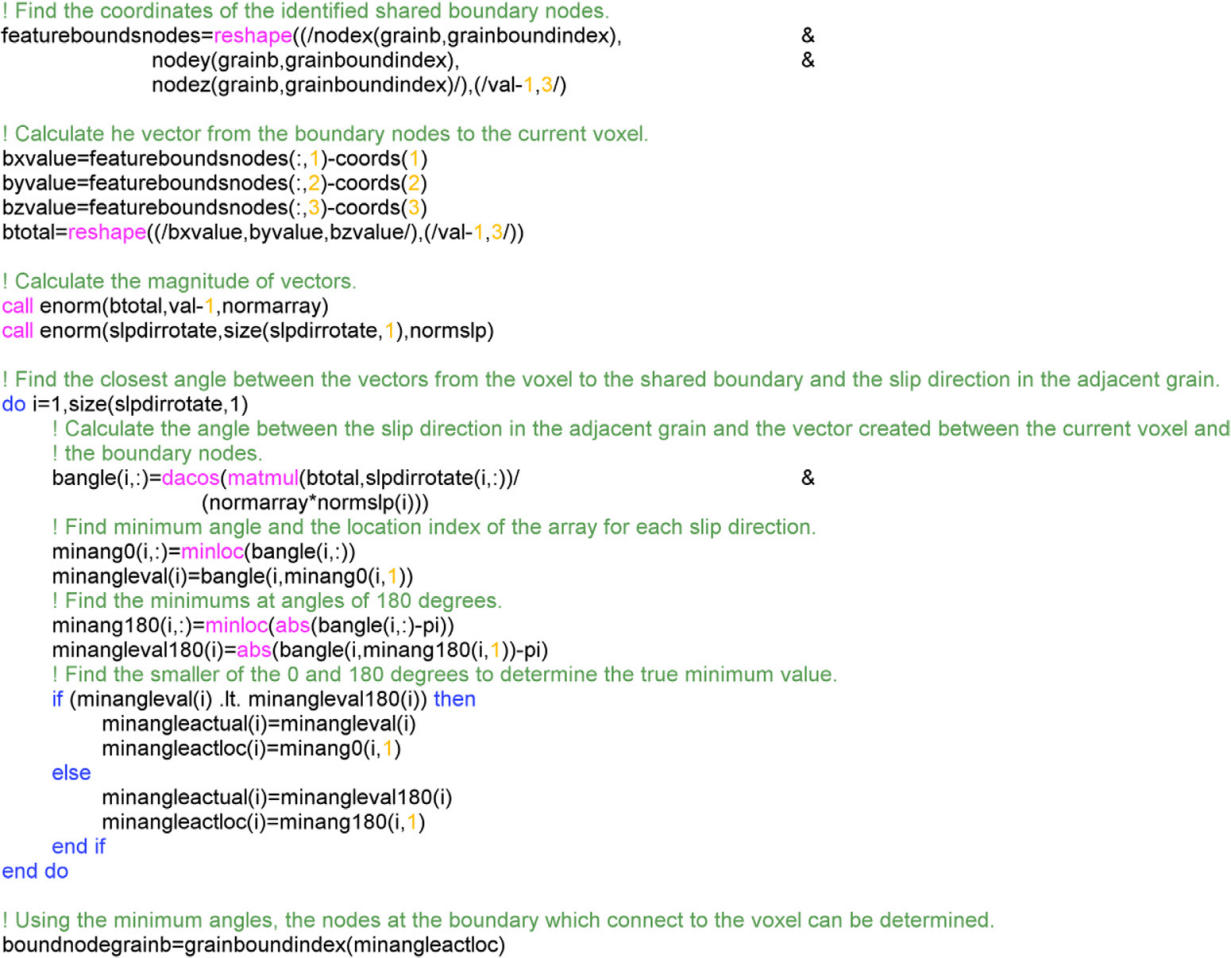
Fortran code to find the nodes at the boundary of grain A and B. This is achieved by finding a vector from the current voxel/element to the boundary which is in the direction of slip in the closest grain.

The source code for how this is implemented is provide in Fig. 9.

**Fig. 10:**
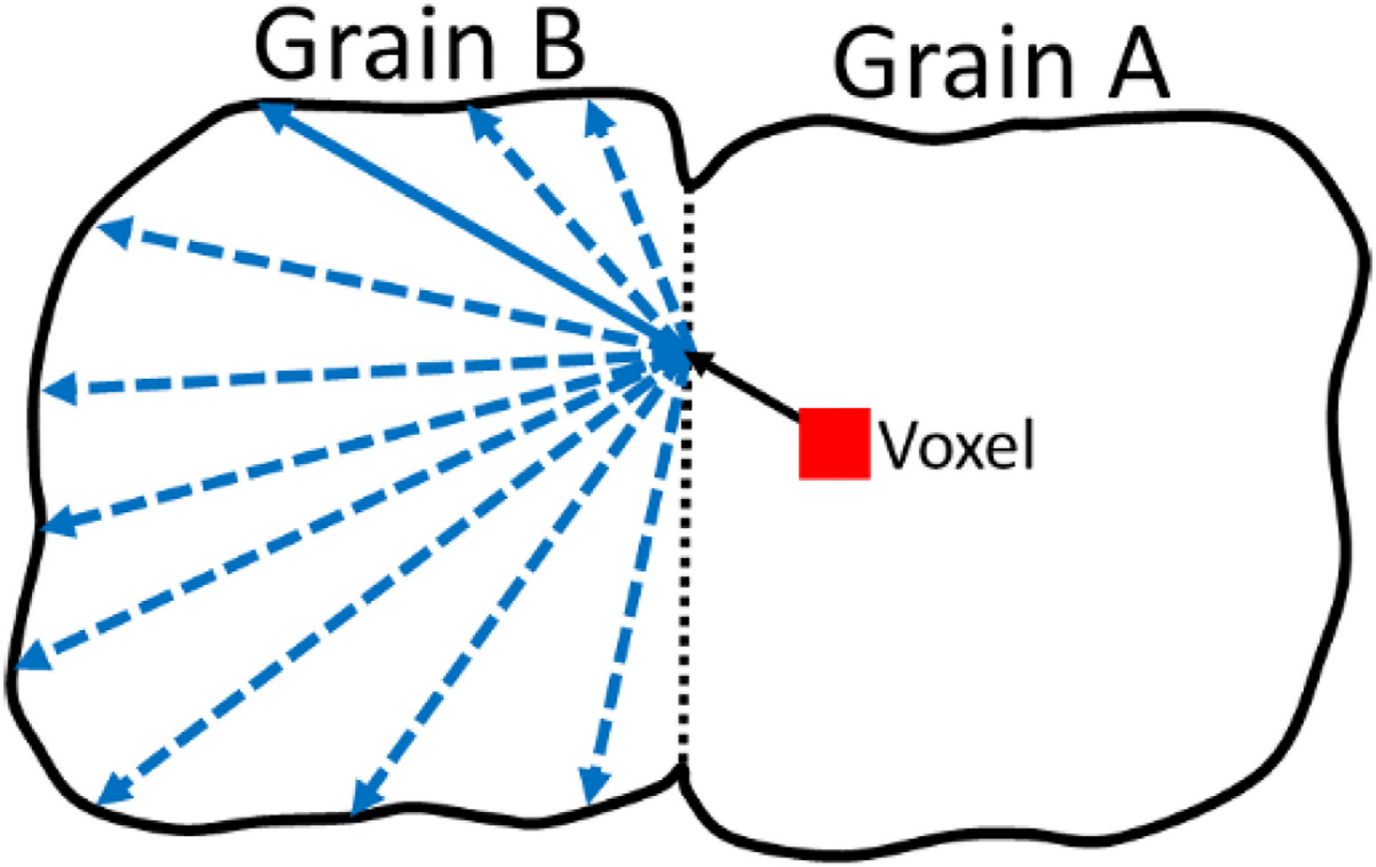
A schematic showing the process from which an appropriately orientated vector is formed in grain B using the identified grain boundary node.

Once the nodes at the shared boundary ($P_{inter}^{\alpha }$) are determined, the next step is to determine the slip length in the adjacent grain. This requires finding the nodes on the boundary of grain B away from the shared boundary which forms a vector orientated in the same direction as slip in this grain B. This process is schematically shown in Fig. 10. In Fig. 10 the vector formed from the voxel/element toward the grain boundary in the direction of slip in grain A ($a_{bound_{k}}^{\alpha }$), is then matched with a vector ($b{{}_{bound}^{\alpha }}_{i}$) formed from the identified point on the grain A-B interface with nodes on the boundary of grain B ($Q_{bound}^{\alpha },\;\text{where}\;Q_{bound}^{\alpha }=\left\{q{{}_{bound}^{\alpha }}_{i}|i\in Z^{+}\right\}$),

(10) $$\vec{P_{inter}^{\alpha }Q_{bound}^{\alpha }}=b{{}_{bound}^{\alpha }}_{i}=Q_{bound}^{\alpha }-P_{inter}^{\alpha },\text{where}\;B_{bound}^{\alpha }=\left\{b{{}_{bound}^{\alpha }}_{i}|i\in Z^{+}\right\}$$

This is done until an appropriately orientated vector is found (given as the vector in bold in grain B).

This process used to determine the nodes on the outer surface of grain B to create the appropriately defined vector ($B_{bound}^{\alpha }$) utilises the same approach as before where the angle between vectors are considered. Firstly, since the vector from the current location (voxel/element) to the boundary nodes is already known, a set of angles $\Theta_{A-B,diff}^{\alpha }$ (where $\Theta_{A-B,diff}^{\alpha }=\left\{\theta {{}_{A-B,diff}^{\alpha }}_{i}|i\in Z^{+}\right\}$) is formed between $a_{bound_{k}}^{\alpha }$ and all vectors within the set $B_{bound}^{\alpha }$,

(11) $$\theta_{A-B,diff}^{\alpha }=\cos^{-1}\frac{a_{bound_{k}}^{\alpha }\cdot b{{}_{bound}^{\alpha }}_{i}}{\left|a_{bound_{k}}^{\alpha }\right|\left|b_{bound_{i}}^{\alpha }\right|}$$

The vectors $a_{bound_{k}}^{\alpha }$and $b{{}_{bound}^{\alpha }}_{i}$ are parallel if the angle between them is 0 or 180°, therefore, the minimum for these two cases are extracted,

(12) $$\theta_{A-B\left(0\right)}^{\alpha }=\min\left(\Theta_{A-B,diff}^{\alpha }\right);\;\theta_{A-B\left(180\right)}^{\alpha }=\min\left(\left|\Theta_{A-B,diff}^{\alpha }-180\right|\right)\;$$

The index of the location of the minimum angles are also stored for later use,

(13) $$\theta_{A-B\left(0\right),index}^{\alpha }=\text{argmin}\left(\Theta_{A-B,diff}^{\alpha }\right);\theta_{A-B\left(180\right),index}^{\alpha }=\text{argmin}\left(\left|\Theta_{A-B,diff}^{\alpha }-180\right|\right)$$

The final minimum angle for each slip system ($\theta_{A-B,min}^{\alpha }$) and therefore the corresponding boundary node can be determined by comparing the two minimums to find the total minimum,

(14) $$\theta_{A-B,min}^{\alpha }=\min\left(\theta_{A-B\left(0\right)},\theta_{A-B\left(180\right)}\right)$$

Using the index of the location within the set at which the minimum is located, the corresponding boundary node ($q_{bound}^{\alpha }$) can be extracted,

(15) $$q_{bound}^{\alpha }=q_{bound\;k}^{\alpha },\;\text{where}\;k=\theta_{A-B\left(0\right),index}^{\alpha }v\theta_{A-B\left(180\right),index}^{\alpha }$$

**Fig. 11:**
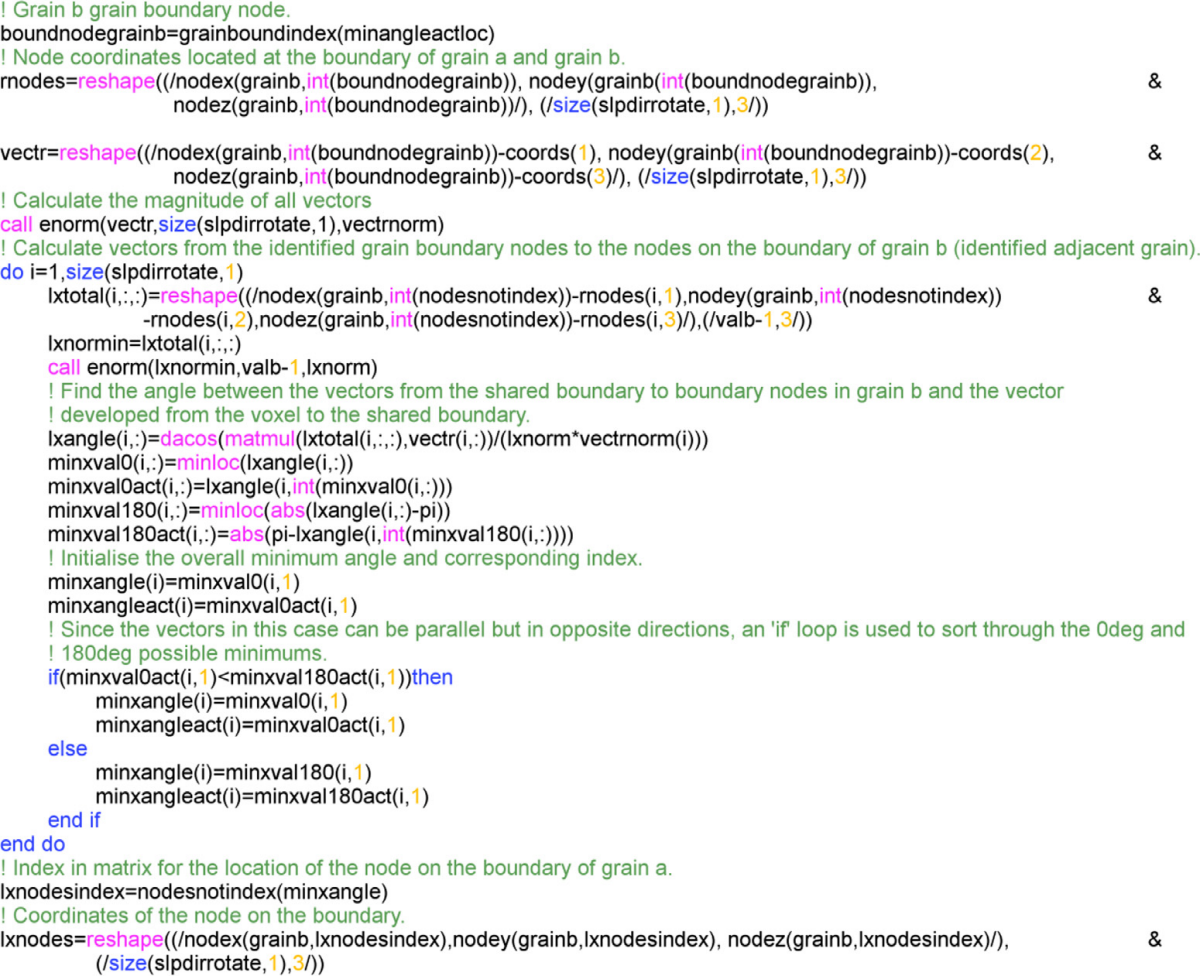
Fortran code used to find the slip distance in grain B from the node at the grain A-B interface.

The source code for how this implemented is provide in Fig. 11.

Once the location on the grain boundary away from the grain A-B interface is determined ($q_{bound}^{\alpha }$), the Euclidean distance from the grain A-B interface node ($P_{inter}^{\alpha }$) to $q_{bound}^{\alpha }$ can be calculated to find the slip distance ($L^{\alpha }$),

(16) $$L^{\alpha }=d^{\alpha }\left(P_{inter}^{\alpha },q_{bound}^{\alpha }\right)=\sqrt{\sum_{k=1}^{n}{\left(q{{}_{bound}^{\alpha }}_{k}-P{{}_{inter}^{\alpha }}_{k}\right)}^{2}}$$

Additionally, the Luster-Morris parameter $\left(m^{\alpha^{\prime }}\right)$ can also be determined using the slip systems in grain A and B,

(17) $$m^{\alpha^{\prime }}=\left(\frac{n_{A}^{\alpha }\cdot n_{B}^{\alpha }}{\left|n_{A}^{\alpha }\right|\left|n_{B}^{\alpha }\right|}\right)\left(\frac{s_{A}^{\alpha }\cdot s_{B}^{\alpha }}{\left|s_{A}^{\alpha }\right|\left|s_{B}^{\alpha }\right|}\right)$$

## Crystal plasticity implementation

The calculation procedure described above is implemented in a Fortran subroutine which can be found in the supplementary information and [12]. This subroutine can be added to crystal plasticity code which can then use the calculated outputs from the subroutine in the underlying constitutive equations, as demonstrated in [1]. The inputs and outputs of this subroutine are listed in Table 2.

**Table 2:** Information on the inputs, where these inputs originate, and outputs for the length-scale subroutine.

Inputs	Description	Origin
Coords	The current voxel/element coordinates which is given as x,y,z values.	This should be available from the software being used since it is the location of the current voxel/integration point at which the calculation is being performed.
Nodeout	Total number of columns for the input matrices (nodex, nodey, nodez, boundfeat), which corresponds to the nodes located on the boundary of every grain.	This is extracted when the matrices are read in at the start of the analysis.
totalfeat	Total number of grains in the RVE.	This is extracted when the matrices are read in at the start of the analysis. Please see the subroutine *arraycoords* in the example Fortran program provided in supplementary material.
nodex, nodey, nodez	x, y, and z coordinates for each node on the boundary of the grains.	Arrays formed from the inputted from the matrices read in at the start of the analysis. Please see the subroutine *arraycoords* in the example Fortran program provided in supplementary material.
slpdir1	Slip directions in the local (crystal) coordinate system	This should be available from information already required for crystal plasticity constitutive models.
slpnor1	Slip plane normals in local (crystal) coordinate system.	This should be available from information already required for crystal plasticity constitutive models.
elcent	Centroid (in x, y, z) of the voxel/element	Array formed from the inputted matrix. Please see the subroutine *el_centroid* in the example Fortran program provided in supplementary material.
feature	The current grain index in which the voxel/element is located.	This should be available from information already required for crystal plasticity constitutive models. If not, this information can be supplied via the inputted material file which is a requirement for all crystal plasticity software.
boundgrain	Total number of boundary grains surrounding each feature (extracted from the inputted multidimensional array boundfeat).	This is extracted when the matrices are read in at the start of the analysis. Please see the subroutine *boundfeat* in the example Fortran program provided in supplementary material.
oriensh	Orientations in Euler angles for each grain within the RVE.	This is information which can be contained in an INCLUDE file (see supplementary information *orien.inc* as an example).
noel	Voxel/element index.	This should be available as a stored value in the software being used since it is the index of the current voxel/integration point.
**Outputs**	**Description**	
rdistance	Distance calculated from the current voxel/element to the grain boundary in the direction of slip in grain A.	
ldistance	Total slip distance in grain B.	
lm	Luster-Morris parameter.	

To demonstrate how the subroutine works, and therefore provide a tool to familiarise potential users, a Fortran program has been developed. This program can be found in the supplementary material and at [12] along with example input data in the form of binary files described in the previous section. The subroutines to read in the binary file matrices are also included in the program (subroutines: *arraycoords, boundfeat, el_centroid*). These subroutines can be copied and used in other crystal plasticity software to initialise the required data at the start of the analysis. If Abaqus is being used, the subroutines *arraycoords, boundfeat, el_centroid* can be added in the subroutine *UEXTERNALDB* which Abaqus calls at the beginning of the analysis, an example of which (*Example_UEXTERNALDB.for*) can be found at [12].

Once compiled, the program will prompt the user to provide a grain ID and element number. Once supplied, the distance of the current voxel/element from the boundary (*rdistance*), slip distance (*ldistance*) in the adjacent grain, and the Luster-Morris parameter (*lm*) will be supplied for each slip system (for this test program, a face centred cubic crystal structure is assumed).

In the developed Fortran program, the subroutine used to generate the slip systems (*slipsysdyn*) was adopted from the classical crystal plasticity subroutine developed by Huang [13].

The advantage of the program is it promotes the development of understanding the intricacies of the presented Fortran subroutine.

## Conclusion

The proposed approach provides the extraction of the slip length in adjacent grains for each slip system. Additionally, the interaction of slip systems in grain A and B are extracted. The Fortran subroutine which implements this approach (found in supplementary documentation) currently uses the interaction of slip systems to calculate the Luster-Morris parameter. However, the extracted difference in orientations can be used to calculate other geometric slip transfer criteria such as those proposed in [14], [15], [16]. Additionally, the distance from the current voxel/element from the boundary in grain A (in the direction of slip in grain B) is also calculated and outputted. This is extra data which can be used in crystal plasticity constitutive models if required.

## Declaration of Competing Interest

The authors declare that they have no known competing financial interests or personal relationships that could have appeared to influence the work reported in this paper.

## Appendix Supplementary materials

Image, application 1

Image, application 2

Image, application 3

Image, application 4
