# Supplementary material for: A method to extract slip system dependent information for crystal plasticity models
Source: MethodsX. 2022 Jun 20;9:101763. doi: 10.1016/j.mex.2022.101763 (PMC9270259; doi:10.1016/j.mex.2022.101763)
Supplement: Supplementary file 4 [file mmc4.docx]

**Additional information on the supplementary data**

Included in the supplementary data are three Fortran files. The differences of these files are as follows:

- grainsize_subroutine.for – This subroutine can be used with Abaqus UMATs. It relies on using a *common block* and additional INCLUDE file (*param_array.inc*) to share the arrays and dimensions from data read in at the start of the analysis (nodex, nodey, nodez, boundgrain, elcent, intotalfeat).
- grainsize_subourtine.f90 – This subroutine does not use a *common block* and relies on the data read in at the start of the analysis (nodex, nodey, nodez, boundgrain, elcent) being an input to the subroutine.
- Lengthscale_program.f90 – Fortran program which can be used to develop a better understanding of the requires of the *grainsize_subroutine*.

All other additional files can be found at the following GitHub repository: https://github.com/DylanAgius/LengMorph.git
